# Endomicroscopy of the Pancreaticobiliary System

**DOI:** 10.1155/2013/310105

**Published:** 2013-02-14

**Authors:** Shajan Peter, Ji Young Bang, Klaus Mönkemuller, Shyam Varardarajulu, C. Mel Wilcox

**Affiliations:** Division of Gastroenterology and Hepatology, University of Alabama at Birmingham, Birmingham, AL 35294-0012, USA

## Abstract

It is often difficult to accurately differentiate between benign and malignant pancreaticobiliary strictures, and some are interpreted as indeterminate despite ERCP, EUS, or radiological imaging techniques, thereby making it difficult for the clinician to make appropriate management decisions. Probe-based confocal laser endomicroscopy (pCLE) is an innovative imaging tool integrating real-time in vivo imaging of these difficult-to-interpret strictures in the pancreaticobiliary system during endoscopy. Recent studies of endomicroscopy have shown a promising role with improved accuracy in distinguishing these lesions, thus paving the way for future research addressing improving precise interpretation, training, and long long-term impact.

## 1. Introduction

**Figure 1:**
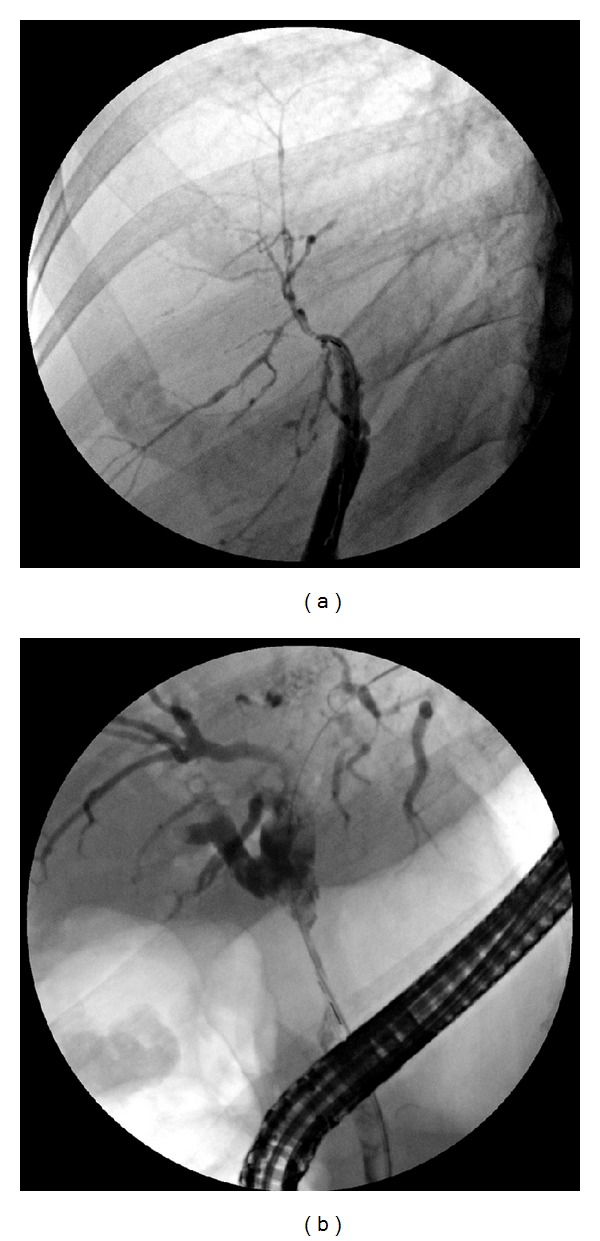
ERCP images of biliary strictures.

Over the past few years, advanced imaging techniques have improved the diagnosis of pancreaticobiliary disorders. While there have been improvements in technology involving procedures such as endoscopic retrograde cholangiopancreaticography (ERCP), endoscopic ultrasound (EUS), computed tomography (CT) scan, magnetic resonance imaging (MRCP), and direct cholangioscopy/SpyGlass, there have also been major advancements in not only diagnosis but also in tissue procurement combined with therapeutic potential. However, in spite of such progress, it remains difficult to accurately differentiate between benign and malignant lesions such as strictures, which are vital for decision-making and appropriate management [1] (Figure 1). Often, conventional methods such as intraductal biopsy, cytological brushings, or FNA remain inconclusive or indeterminate resulting in low diagnostic accuracy [2] (Figure 5). These scenarios lead to further testing or intervention; delay diagnosis is important for a potential candidate requiring surgical resection or palliative therapy. Probe-based confocal laser endomicroscopy (pCLE) is one innovative tool that allows for real-time in vivo imaging of these difficult-to-interpret strictures in the pancreaticobiliary system.

Current diagnostic modalities (biopsy, brush cytology, or fine needle aspiration (FNA) through ERCP or EUS guided routes) have their limitations in accurate diagnosis of biliary strictures, and the sensitivity of tissue sampling varies between 20% and 60% [3–5]. The sensitivity increases when two sampling methods are utilized such as combining brush cytology with forceps yielding a sensitivity ranging from 54% to 70.4% and specificity from 97% to 100% [6]. Adding EUS-guided FNA to these methods increased the sensitivity marginally to 71% with specificity of 100%, though the EUS technique is the best method for diagnosing intrapancreatic lesions. In a recent study, standard ERCP-guided tissue sampling in indeterminate biliary strictures had 76% sensitivity and 88% accuracy, while these values were 57% and 78%, respectively, for cholangioscopic-directed (Spy Bite) biopsies [7]. These data show that given the best available techniques, the yield is still suboptimal. The site of stricture such as proximal versus distal, nature of sampling, superficial versus deep, and difficulty in focal targeting add to the existing challenges facing the diagnostic dilemma while evaluating indeterminate biliary strictures thereby underlying the dire need for improving overall accuracy.

Confocal laser endomicroscopy (CLE) has emerged as a new endoscopic imaging modality helping the endoscopist become an endopathologist by obtaining in vivo histological assessment. Thus it offers an additional or alternative method to tissue “sampling” in assessing strictures. There are two commercially available systems: (a) the endoscope-based CLE system where the confocal mechanism is incorporated in the tip of a conventional endoscope, integrated CLE (iCLE), and (b) the probe-based CLE (pCLE) system that can be passed through the biopsy channel of the endoscope. For real-time imaging of the biliary system owing to accessibility and delivery the latter system is currently being used [8].

The pCLE probe that is FDA approved specifically for pancreaticobiliary use is the CholangioFlex^UHD^ miniprobe (Mauna Kea Technologies, Paris, France) [9]. This probe consists of several fiber light bundles (>10000 optical fibers) with distal lens through which the laser beam is transmitted while being connected to a laser-scanning unit and light source. The diameter is smaller than a GastroFlex^UHD^ miniprobe, which is used for another upper gastrointestinal imaging. The specifications are listed in Table 1.

The CLE images are then sent to a laser scanning unit (LSU) that interprets the images and sends them to a processor. Data are collected at 12 frames per second. These images and acquired sequences are stored on computer systems, where they can be viewed and interpreted as real time or recorded and saved. They can be then reviewed, magnified, or exported. The specially designed software package (Cellvizio viewer) allows image correction and stabilization. 

## 2. Contrast Agents

Exogenous fluorescent agents are needed for detection of cellular details while performing a CLE test. Intravenous fluorescein sodium is commonly used at 10% concentration, which then helps in highlighting vessels, intercellular spaces, vascular pattern, lamina propria, and overall cellular architecture. It does not stain nuclei. Thus, disruption of cellular architecture or vasculature or leaky blood vessels are indicative of disease. When used mostly by ophthalmologists fluorescent agents have shown a high safety profile, and adverse events are few [10]. One must caution the patient that they may experience transient yellowing of the skin, eyes, and urine that could persist for a few hours. Typically an injection of 2.5 to 5.0 mL of fluorescein is sufficient for visualization of epithelial cells, and the effect lasts for 30 minutes.

## 3. Technique

The pCLE probe is inserted through the working channel of an ERCP scope or through various catheter devices (Table 2). It is advanced until the radiopaque tip is visible under fluoroscopic vision or cholangioscopic visualization. It is then positioned in direct contact with the mucosa of the biliary tract or site of interest. It might be preferred to go from an area of normal mucosa to abnormality. After injecting fluorescein intravenously, using the foot pedals provided or from the computer screen activates the laser, and real-time endomicroscopy is performed. Given the narrowing of lumen specially while imaging biliary duct strictures, the main challenge is positioning the probe to maintain it perpendicular to the mucosa rather than parallel. The video sequences and images can be captured or recorded. Tissue sampling such as for cytology or biopsies are preferably done after image acquisition by pCLE as they could result in art factual errors. Access of the pCLE probe can also be achieved through the SpyGlass system (Boston Scientific, Natick, Massachusetts, USA), Olympus cholangioscopes (Olympus Corp, Tokyo, Japan), and Storz prototype cholangioscope (Karl Storz GmbH, Tuttlingen, Germany) [17].

## 4. Interpretation

**Figure 2:**
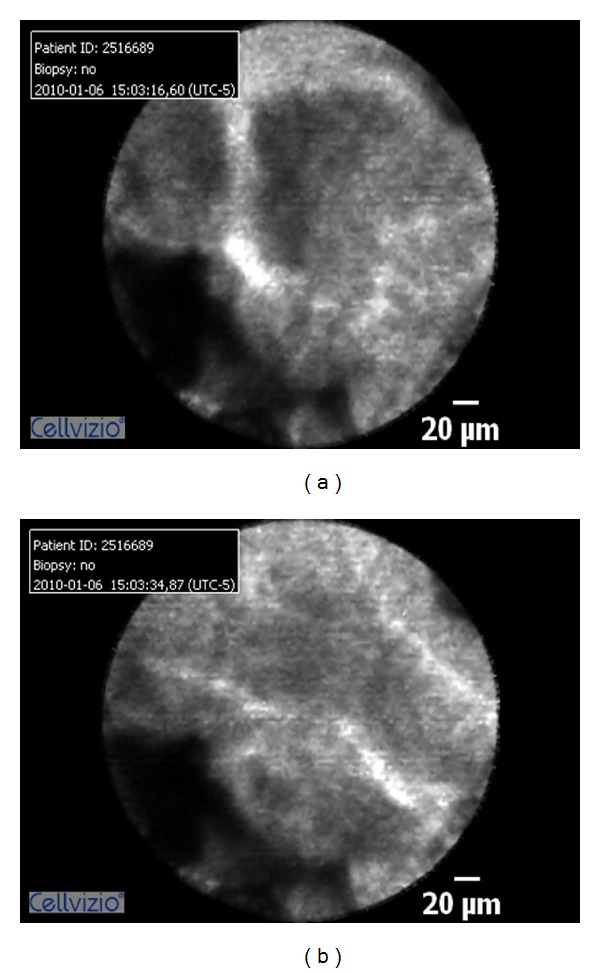
Normal appearing bile duct with fine, reticular pattern.

**Figure 3:**
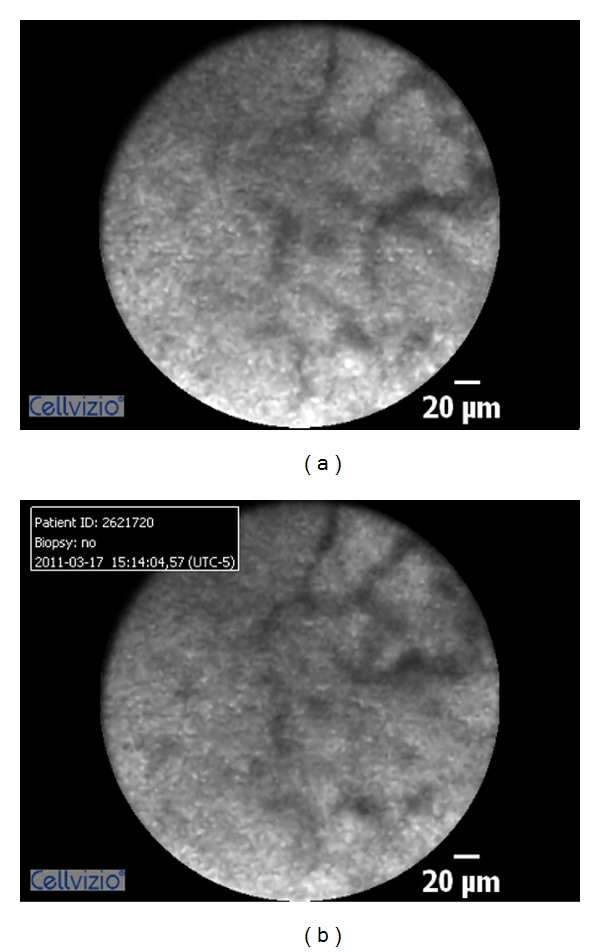
Normal appearing bile duct and reticular pattern.

**Figure 4:**
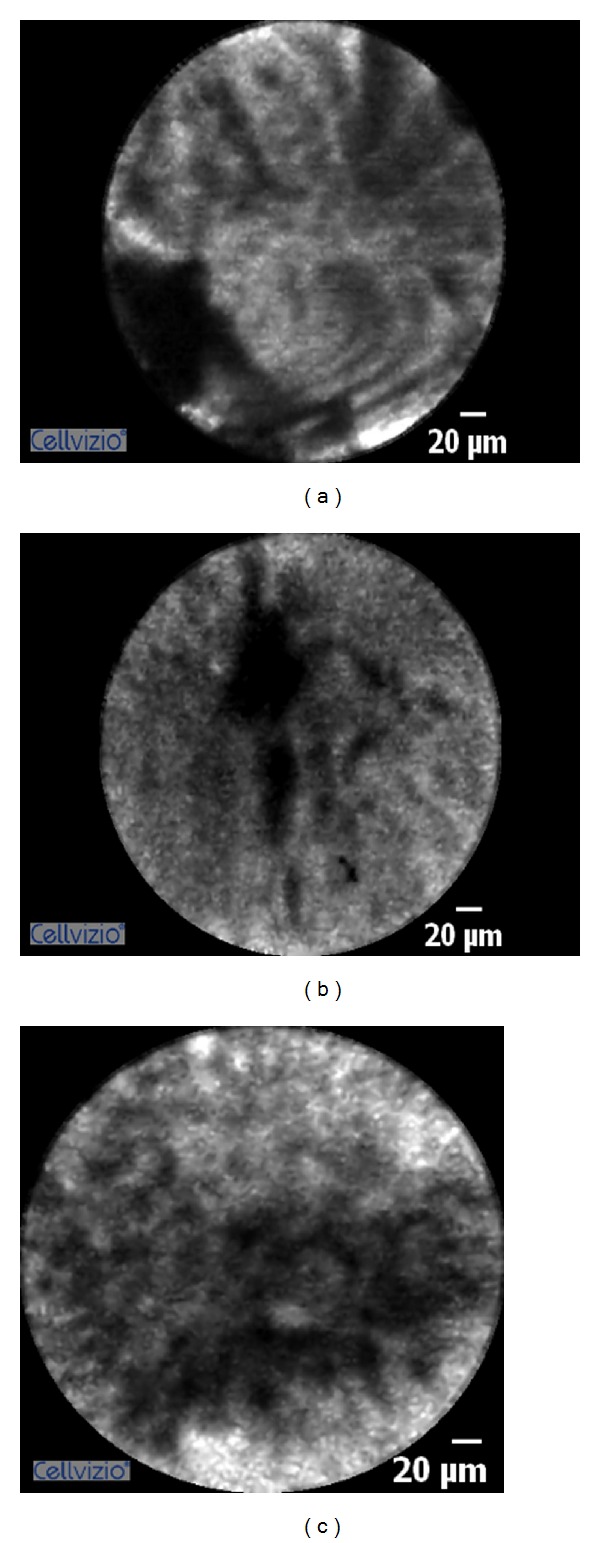
Biliary malignancy showing features of dark irregular structures, thick bands >20 *μ*m, tortuous dilated blood vessels, and areas of dark clumps.

**Figure 5:**
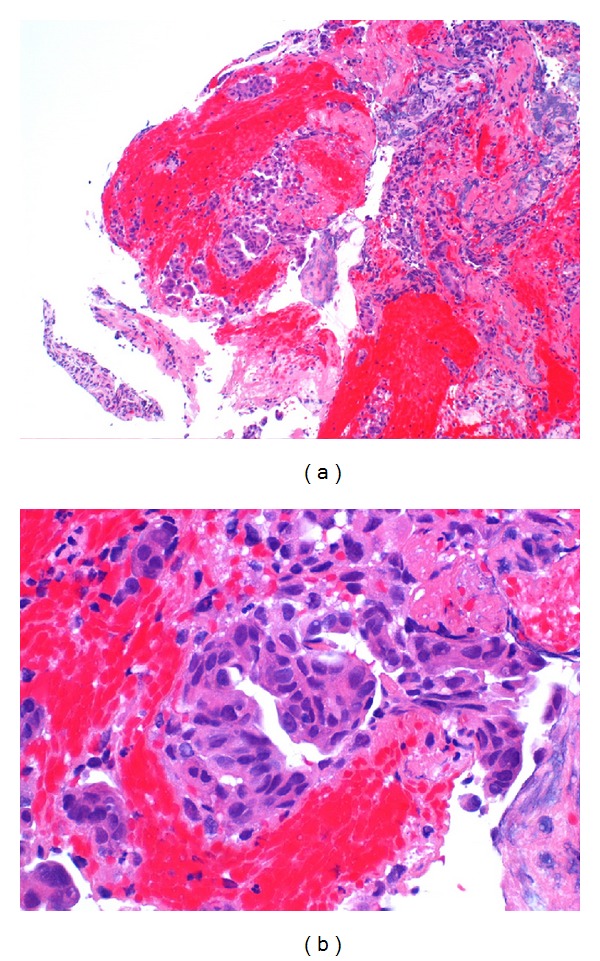
Cytopathology images of malignant biliary strictures (a) 10x resolution and (b) 40x resolution.

Features that differentiate benign mucosa from malignant mucosa include loss of the reticular pattern of epithelial bands <20 *μ*m, irregular epithelial lining, gland-like structures, tortuous dilated vessels with abnormal branching, and clumps of black areas showing focal decreased areas of fluorescein or fluorescein leakage (Figures 2, 3, and 4). Putting all these observations together, the Miami classification has been adopted for classification of biliary and pancreatic lesions especially for indeterminate strictures [18]. Using specific criteria such as thick white bands (>20 *μ*m), or thick dark bands (>40 *μ*m) or dark clumps and epithelial structures, resulted in sensitivity of 97%, specificity (33%), PPV (80%), and NPV, (80%) compared with 45%, 100%, 100%, and 69%, respectively, for standard cytopathology. The interobserver variability was moderate for most criteria. In retrospective analysis, combining two or more criteria increased the sensitivity as compared to single criteria interpretation.

## 5. Studies

Few studies have evaluated the role of pCLE in evaluating pancreaticobiliary strictures (Table 3). Shieh et al. used the GastroFlex miniprobe for CBD evaluation and noted that cellular architecture was better visualized using this probe as compared to the CholangioFlex probe with no major side effects [13]. Giovannini et al. used the Cholangioflex probe in 37 patients who had ERCP for bile duct stones or stenosis and interpreted images in 33 [15]. Their study predicted an accuracy of 86%, sensitivity of 83%, and specificity of 75% compared to routine biopsies with respective values of 53%, 65%, and 53%. Meining et al. conducted a multicenter study in 89 patients with indeterminate biliary strictures using the CholangioFlex probe with a one-month followup after the procedure [19]. They were able to predict malignancy in 40 patients that was subsequently confirmed on followup. pCLE had a sensitivity of 98%, specificity of 67%, and accuracy of 81% as compared to 45%, 100%, and 75%, respectively, for index pathology. Accuracy for combination of ERCP and pCLE was also significantly higher compared with ERCP and tissue acquisition (90% versus 73%). Also of interest, there was no improvement in pCLE diagnostic accuracy during the course of the study suggesting a short learning curve. Loeser et al. evaluated a smaller sample of 14 patients with indeterminate strictures of bile ducts using both the Cholangioflex and occasionally the GastroFlex probes [14]. They pointed to a normal reticular network pattern in benign lesions and comparison was made to multiphoton reconstructions of intact rat bile ducts [16]. The similarly observed reticular pattern corresponded to lymphatic structures, and presumably distortion of this pattern could be occurring in malignancy. Other criteria such as dilated blood vessels were not specific as they were observed in both benign and malignant strictures such that the negative predictive value suggests ruling out a malignancy.

## 6. Limitations and Promising Role

These studies shed light on the emerging role of pCLE for evaluation of indeterminate biliary strictures although limited by small sample size and specific population subsets. The diagnostic criteria are still evolving, and the exact anatomic correlates that result in the observed “abnormal” features remain undefined. One might speculate that white bands reflect blood vessels and increased neovascularization. Black bands could resemble lymphatic vessels, and the “clumps-” like structures correspond to tissue proliferation patterns, or these could mirror a paraneoplastic phenomenon. Observations such as the mucosal band and clump thickness are visual estimates and therefore subject to interpreter error. Also it is yet not clear which of these findings are specific to biliary versus pancreatic malignancies. In many of these studies, the endoscopists were aware of the clinical history and ERCP/CT or MRI findings while interpreting the imaging resulting in an interpreter bias. In a recent multicenter study, twenty-five deidentified pCLE video clips of indeterminate biliary strictures were sent to 6 observers at 5 institutions. Using the Miami Classification for standard image interpretation, they concluded that overall interobserver agreement with Kappa scores was poor to fair [20]. Further, the choice of optimal access delivery catheter versus cholangioscopic is still not clear and needs to be investigated, as well as the difference between using a larger diameter probe (Gastroflex) from the miniprobe (Cholangioflex) keeping in mind the respective spatial resolution and image quality. These factors may be important while manipulating and targeting those challenging malignant lesions in the bile duct especially when growth occurs longitudinally along the wall rather than an intraluminal ingrowth manner. The cost effectiveness of pCLE is yet to be determined as it is not clear whether this tool is sufficient enough to replace histology. Nevertheless, it could prevent repeated endoscopic evaluations with attempt at biopsies in indeterminate cases. The possibilities of use during laparoscopy or laparotomy identifying disease-free margins are other potential areas where pCLE might play an expanding role.

## 7. Future

Clearly, further studies are needed before pCLE is adopted as an integral technique for evaluation of pancreaticobiliary disease. Future studies should focus on prospective validating while defining one or more specific criteria for pancreaticobiliary disorders ranging from normal to inflammatory, early neoplastic, to disease specific such as cholangiocarcinomas, gallbladder malignancies, infiltrative pancreatic cancers, and primary-sclerosing-cholangitis-(PSC-) related tumors. Modification of probes for better image stability and resolution will be technical innovative challenge. Use of EUS-guided FNA needles is being studied and could potentially help to reach territories such as pancreatic cysts. Molecular imaging using fluorescent-tagged peptides developed from bacteriophage libraries selective to biliary tissues focuses on newer areas of research [21]. These peptides could then be detected by CLE and differentiate between benign and dysplastic/neoplastic tissues.

## 8. Conclusions

The newer endoscopic imaging modality of CLE has kindled an interest in the field of advanced imaging offering real-time histopathologic evaluation of the pancreaticobiliary system. It has strengthened and extended the arm of the gastroenterologist from a therapeutic endoscopist to an endopathologist. The novel use of this technique is particularly of significance in differentiating indeterminate biliary strictures as treatment depends on an accurate and prompt diagnosis. More studies will ultimately determine its precise role in the immediate or long-term impact as well in combination with available modalities in the treatment of pancreaticobiliary disorders.

## Figures and Tables

**Table 1:** Specifications of pCLE probe for pancreatico-biliary evaluation.

	Mini probe (CholangioFlex)
Laser wavelength	488 nm
Depth of imaging (*μ*m)	40–60
Plane depth (*μ*m)	0–50
Max field of view (mm)	325
Lateral resolution (*μ*m)	3.5
Axial resolution (*μ*m)	15
Imaging rate (frames/s)	12
External diameter (mm)	0.94

**Table 2:** Type of catheters for pCLE access.

Catheter device	Manufacturer
Cotton Graduated Dilatation Catheter	Cook Medical
OASIS One Action Stent Introduction System	Cook Medical
Memory Dormia Basket	Cook Medical
Howell Biliary Introducer	Cook Medical
Geenen Graduated Dilatation Catheter	Cook Medical
Swing Tip ERCP Cannula	Olympus Medical

**Table 3:** Relevant studies for pCLE of the biliary system [11].

Study (year)	Number of patients	Number malignant	Specificity	Sensitivity	Accuracy
Meining et al. [12] (2008)	14	6	88	83	86
Shieh et al. [13] (2012)	11	NA	NA	NA	NA
Loeser et al. [14] (2011)	14	6	NA	NA	NA
Giovannini et al. [15] (2011)	37	23	75	83	86
Meining et al. [19] (2011)	89	40	67	98	81
